# The fairness, predictive validity and acceptability of multiple mini interview in an internationally diverse student population- a mixed methods study

**DOI:** 10.1186/s12909-014-0267-0

**Published:** 2014-12-21

**Authors:** Maureen E Kelly, Jon Dowell, Adrian Husbands, John Newell, Siun O‘Flynn, Thomas Kropmans, Fidelma P Dunne, Andrew W Murphy

**Affiliations:** The Medical School, National University of Ireland Galway, (NUI Galway), Galway, Ireland; School of Medicine, University of Dundee, Dundee, Scotland; Health Research Board (HRB) Clinical Research Facility, National University of Ireland, Galway (NUI Galway), Galway, Ireland; The Medical School, University College Cork (UCC), Cork, Ireland

**Keywords:** Multiple mini interview, Selection, International students, Mixed methods, Culture, Language, Recommendations, Stakeholder views

## Abstract

**Background:**

International medical students, those attending medical school outside of their country of citizenship, account for a growing proportion of medical undergraduates worldwide. This study aimed to establish the fairness, predictive validity and acceptability of Multiple Mini Interview (MMI) in an internationally diverse student population.

**Methods:**

This was an explanatory sequential, mixed methods study. All students in First Year Medicine, National University of Ireland Galway 2012 were eligible to sit a previously validated 10 station MMI. Quantitative data comprised: demographics, selection tool scores and First Year Assessment scores. Qualitative data comprised separate focus groups with MMI Assessors, EU and Non-EU students.

**Results:**

109 students participated (45% of class). Of this 41.3% (n = 45) were Non-EU and 35.8% (n = 39) did not have English as first language. Age, gender and socioeconomic class did not impact on MMI scores. Non-EU students and those for whom English was not a first language achieved significantly lower scores on MMI than their EU and English speaking counterparts (difference in mean 11.9% and 12.2% respectively, P<0.001). MMI score was associated with English language proficiency (IELTS) (r = 0.5, P<0.01). Correlations emerged between First Year results and IELTS (r = 0.44; p = 0.006; n = 38) and EU school exit exam (r = 0.52; p<0.001; n = 56). MMI predicted EU student OSCE performance (r = 0.27; p = 0.03; n = 64). In the analysis of focus group data two overarching themes emerged: Authenticity and Cultural Awareness. MMI was considered a highly authentic assessment that offered a deeper understanding of the applicant than traditional tools, with an immediate relevance to clinical practice. Cultural specificity of some stations and English language proficiency were seen to disadvantage international students. Recommendations included cultural awareness training for MMI assessors, designing and piloting culturally neutral stations, lengthening station duration and providing high quality advance information to candidates.

**Conclusion:**

MMI is a welcome addition to assessment armamentarium for selection, particularly with regard to stakeholder acceptability. Understanding the mediating and moderating influences for differences in performance of international candidates is essential to ensure that MMI complies with the metrics of good assessment practice and principles of both distributive and procedural justice for all applicants, irrespective of nationality and cultural background.

**Electronic supplementary material:**

The online version of this article (doi:10.1186/s12909-014-0267-0) contains supplementary material, which is available to authorized users.

## Background

International medical students, those who attend medical school outside of their country of citizenship, account for a significant proportion of medical school undergraduates [1]. For example they make up 7.5% of medical school undergraduates in the UK [2], and account for over 15% of Australian medical graduates [3]. International medical graduates (IMGs) account for approximately 25% of practicing physicians in the USA, with returning USA citizens accounting for a growing proportion of these [4]. According to the OECD the number of students migrating in pursuit of higher education is on the increase rising from 0.8 million worldwide in 1975 to 4.3 million in 2011 [5].

Interview remains a stalwart of medical student selection internationally [6,7]. A survey of First Year medical students in Ireland (n = 291) revealed that over 70% were in favour of selection interviews with international students significantly more likely to hold this view (p ≤ 0.001) [8]. Multiple Mini Interview is the most robust structured interview in use today; avoiding many of the shortcomings of the traditional interview [9,10]. A recent systematic review of MMI specifically called for research to determine its acceptability in different cultural contexts which has particular relevance for international medical school applicants [11].

There is limited exploration of the challenges of international medical selection in the literature, with existing research largely restricted to postgraduate professional training. There is controversy with respect to the performance of IMGs in postgraduate examinations and progression on higher professional training programmes. Three recent BMJ articles highlight the complexities and sensitivities in this area particularly with regard to issues of fairness and equivalence, issues which are also relevant to selection [12-14].

Stakeholder opinion is an important consideration in the *political validity* of selection tools [6]. In one study of paediatric higher professional training in the UK international graduates were more likely than UK graduates to prefer MMI over traditional interview as a selection tool (p = 0.01) [15]. A study of Canadian graduates and IMGs, applying to postgraduate training, found that MMI was considered reasonable by 88% of candidates and 90% of assessors although there was no differentiation of the views of Canadian and IMGs, nor were assessors’ views reported [16]. Patterson et al. established the usefulness of *organisational justice theories* to conceptualise stakeholder reactions to selection tools in medicine [17,18]. Two of these theories are particularly relevant: *Distributive justice* relates to the fairness of the selection *outcome* - such as securing a medical school place, in terms of equal opportunity and equity. *Procedural justice* relates to the fairness of the selection *process* in terms of job relevance, characteristics of the test and interpersonal treatment [17]. These values complement the criteria expected of selection tools as defined by the Ottawa consensus statement, which conceptualised selection as “*assessment for selection*” and recommended that the principles of good assessment be applied to selection tools [19].

The five undergraduate medical schools in Ireland host a significant number of international students annually. MMI is not routinely used in the selection of medical students in Ireland. However a pilot study in 2012 established the feasibility of running a MMI in an Irish setting [20].

The aims of this study were to run an experimental MMI in an internationally diverse student population to establish its

- Fairness with respect to age, gender, socioeconomic group and candidate background,
- Predictive validity in year one assessment outcomes,
- Stakeholder (MMI candidates and assessors) acceptability.

With respect to selection the term fairness implies that everyone has a fair opportunity and chance of being selected based on talent and merit alone [21]. Predictive validity determines how well scores on a selection measure predicts some future outcome, such as work performance or examination scores [6]. Stakeholder acceptability describes the views of those who are affected by or can affect selection processes, such as applicants, employers, parents and the regulator, on the suitability and appropriateness of the tool for use in selection [22,6]. In this paper we discuss stakeholders’ views of the acceptability of MMI through the lens of organisational justice theory.

This study was set in the School of Medicine, National University of Ireland, Galway an undergraduate medical school with over 1000 students, in collaboration with the Medical School Dundee (UK) and University College Cork, Ireland.

The selection criteria for medical students in Ireland are determined by whether their country of origin is within or out with the European Union (EU) (see Table 1). EU school leavers apply via a single national system administered by the Central Applications Office (CAO) and are selected on the basis of academic record and score in an adjunct admission test -HPAT-Ireland, with an additional requirement for an English language test if prior academic record has not included English as a subject [23-25]. There is no national protocol for selection of Non-EU students however most schools require academic record, evidence of English language proficiency and interview. Non-EU applicants are not required to sit HPAT-Ireland. The most commonly used test of English proficiency is the International English Language Testing System (IELTS) [26]. This test comprises four domains; Listening, Reading, Writing and Speaking. At present in Ireland the minimum acceptable IELTs score is 6.5.

**Table 1:** **Criteria for medical student selection in Ireland**

**EU Applicants Selection Criteria**	**Non-EU applicants Selection Criteria**
Academic Record: Leaving Certificate Examination or equivalent	Academic Record: Grade Point Average
English Language Proficiency - if required	English Language Proficiency: International English Language Testing System (IELTS) or equivalent
Health Professions Admission test – Ireland (HPAT-Ireland)	+/- Traditional Interview
	+/- Others including MCAT, Personal statement, reference

## Methods

The research paradigm was mixed methods, adopting a pragmatic worldview [27]. Mixed methods have been identified as an appropriate design for studying complex medical education topics and are “*increasingly relevant to medical education”* [28,29].

An explanatory sequential study design entailed conducting a quantitative strand first (MMI- see below) followed by a qualitative strand (focus group interviews- see below). Data from both strands were analysed independently then considered together in the subsequent interpretation of findings. In general the quantitative data evaluated the predictive validity and fairness of the MMI while the qualitative data explored acceptability. Equal weighting was given to both sources of data (QUAN → QUAL) [30]. Ethical approval was granted by NUI Galway Research Ethics Committee. Written consent to participate in the study was obtained from students who sat the MMI and additionally from all focus groups participants.

### Quantitative strand

All students enrolled, in First Year Medicine, NUI Galway, September 2012 were invited to participate. Volunteers were entered into a raffle for an iPad. A previously validated MMI was used (Additional file 1: Appendix 1). It consisted of ten, 7 minute stations. Full details of station breakdown, blueprinting of stations, assessor recruitment & training and student and assessor feedback are previously published [20]. Cronbach’s Alpha of MMI items was 0.78.

Post MMI, students were provided with individual written feedback highlighting their three best and weakest performing stations and information on how each station mapped to the eight domains of professional practice as per the Irish Medical Council. [31].

Demographic data collected were student age, gender, nationality, first language and parental occupation. EU student selection data comprised school exit exam (predominately Leaving Certificate Examination-LCE) and HPAT-Ireland score. Non-EU student selection data comprised IELTS score, Grade Point Average (GPA) and Interview score. Experimental data consisted of MMI scores which were collected for all participants.

Outcome variables were First Year examination results (Additional file 1: Appendix 2) comprising First Med Score (a continuous variable representing each student’s overall performance on the First Year Examinations) and First Med OSCE score (a continuous variable representing performance on a five station OSCE assessing communication and clinical skills).

Quantitative data were entered into PASW (formerly SPSS) [32]. We report descriptive statistics, student t tests, chi-square, Pearson’s product moment correlation coefficient, Spearman’s Rho and regression analysis. Strength of correlations were compared using Cohen’s effect size interpretations (small ≥ .10, medium ≥ .30, large ≥ .50) [33].

### Qualitative strand

Focus groups were utilised as they are effective in accessing a broad range of views; offer participants an opportunity to consider their own views in the context of others and are particularly appropriate for culturally sensitive issues [34-36]. All student participants and MMI assessors were invited by email to take part in focus group interviews. Four separate homogenous focus groups were conducted in order to capitalise on people’s shared experiences; two MMI Assessor focus groups (7 and 6 participants respectively), one EU student (7 participants) and one non-EU student focus group (8 participants) [36].

Each focus group lasted approximately an hour and was conducted on campus, by an independent experienced moderator. A second researcher took field notes and attended to flow. The topic guide was based on a post MMI evaluation questionnaire administered in the feasibility study [20] (Additional file 1: Appendix 3). Focus groups were audiotaped and transcribed verbatim. Debriefing took place between the focus group moderator, note taker and one of the authors (MK). Field notes were used to clarify and add contextual details to the transcribed interviews [34]. Transcripts were independently open coded by three authors (AH, AWM, MK); codes were compared and discussed until agreement was reached. Axial and selective coding took place in an iterative fashion using the constant comparison technique. The final themes were agreed upon by all authors. N-Vivo10 software was used [37].

### Quality and rigour

To help ensure reflexivity a coding diary recorded reflections on how the researchers’ own experiences may have shaped the collection, generation and analysis of data [38]. Steps were taken throughout the research process to “bracket” any prior assumptions and experiences [39]. For example researchers were mindful that knowledge of the quantitative findings may influence their subsequent interpretation of focus group data and through careful re-reading of the data they sought to become aware of and manage these influences. Care was also taken to understand “deviant” or “negative” views and to actively look for opinions and thoughts that ran contrary to the researcher’s own opinions. With respect to “fair dealing”, attention was paid to not over emphasising the views of any one group of participants as if they represented the sole truth [36].

## Results

### Quantitative results

#### Descriptive statistics

There were 241 eligible students in the year (54% (n = 130) Female; 43% (n = 103) Non-EU). Of these 109 (45%) students participated of which 62.4% were female (n = 68) see Table 2. Mean age was 19.64 years (SD 1.3; 95% CI 19.4-19.9 years). Of the sample 58.7% (n = 64) were EU origin and 41.3% (n = 45) were Non-EU. Similar proportions of EU and Non-EU students volunteered for the study (44% and 46% respectively). English was the first language of 64.2% (n = 70). Socioeconomic Class (SEC) was based on parental occupation as per Census 2011 guidelines with specific advice on applying these guidelines to Malaysian students sought from a medical academic in Kuala Lumpar [40,41].

**Table 2:** **Participants’ demographics**

	**Total sample (100%, n = 109)**	EU origin* (58.7%, n = 64)	Non-EU origin** (41.3%, n = 45)
**Age**	Mean 19.64 yrs (SD 1.32; 95% CI 19.39-19.89)	Mean age = 19.5 yrs (SD 1.26)	Mean age = 19.84 yrs (SD 1.38)
**Gender**	Female 62.4% (n = 68)	Female 50% (n-32)	Female 80% (n = 36)
	Male 37.6% (n = 42)	Male 50% (n = 32):	Male 20% (n = 9):
**Speaks English as first language**	64.2% (n = 70)	96.9% (n = 62)	17.8% ( n = 8)
**Socioeconomic class**			
SEC 1 = Professional workers	33% (n = 36)	SEC 1& 2 combined	SEC 1& 2 combined
		67% (n = 43)	84% (n = 38)
SEC 2 = Managerial and technical	41% (n = 45)		
SEC 3 = Non-manual	16% (n = 17)	SEC 3, 4, 5 combined	SEC 3, 4, 5 combined
SEC 4 = Skilled Manual	3% ( n = 3)	27% (n = 17)	16% (n = 7)
SEC 5 = Semi skilled	4% (n = 4)		
Missing data for SEC	4% (n = 4)	6% (n = 4)	0% (n = 0)

Footnote *The EU group comprised 61 from Ireland (56% of the overall cohort) and 1 (0.9%) each from Great Britain, Finland and Germany. **The Non-EU group comprised 37 from Malaysia (33.9%), 5 from Singapore (4.6%); 2 from Canada (1.8%), and 1 (0.9%) from USA).

### Between group comparisons

Between groups comparisons revealed no statistical difference between mean age of the EU and Non-EU students (T-test (two tailed) p = 0.18) nor the proportion of students in SEC 1 & 2 and SEC 3, 4, 5 in the different groups (Chi Square p = 0.19 (df = 1)). There was a significantly higher proportion of females and students who did not have English as a first language in the Non-EU group (Non-EU group Females 80% (n = 36): EU students Females 50% (n = 32) Chi Square p = 0.003 (df = 1): Non-EU group with English as a First Language 17.8% (n = 8): EU group with English as First Language 96.9% (n = 62) Chi Square p < 0.01 (df = 1)). These differences reflected the First Year Medical class norms.

### Selection and experimental data

Table 3 presents the selection tool scores, including MMI, and indicates the relationship between candidates’ scores, gender, SEC and age. Mean MMI score 67.1% (n = 109) SD 9.7, 95% CI 65.2-68.9. EU students scored significantly higher on MMI than Non-EU students (EU (n = 64) mean 72.0%; Non-EU (n = 45) mean 60.1%; p < 0.001; Difference in mean 11.9%, 95% CI 8.8-14.9). Likewise students with English as a first language scored significantly higher on MMI (First Language English (n = 70) mean MMI 71.4%; First language not English (n = 39) mean MMI 59.2%; p < 0.001; Difference in mean 12.2%; 95% CI 9.0-15.4).

**Table 3:** Predictor variables mean scores and relationship with gender, socioeconomic group and age*

**Selection tool**	**Mean score (SD, 95% CI for the mean)** ***except where noted***	**Female versus Male scores**	**SEC 1&2 versus SEC 3,4,5 scores**	**Correlation with Age** ***(Pearson’s product moment correlation- except where noted)***
**LCE**	98.8% (n = 56) SD = 0.7; 95% CI 98.6-99.0%	Female 98.7% (n = 26)SD 0.7; Male 99% (n = 30) SD 0.7 p = 0.59	SEC 1&2 98.8% (n = 40) SD 0.7; SEC 3, 4, 5 98.9% (n = 12) SD 0.7 p = 0.83	***r = −0.28 (n = 56) p = 0.04***
**HPAT-Ireland**	185.2 (out of max possible 300) (n = 63) SD 10.1, 95% CI 182.6-187.7	Females (n = 31) 184.6; Males (n = 32) 185.8 p = 0.64	SEC 1&2 (n = 43) 186.2; SEC 3, 4,&5 (n = 16) 182.7p = 0.19	r = −0.03 (n = 64) p = 0.79
**IELTS**	7.2 (n = 38) SD 0.5; 95% CI 7.0-7.3	Females (n = 31 ) 7.2; Males (n = 7) 7.1, p 0.98	SEC 1&2 (n = 81) 7.2; SEC 3, 4, 5, (n = 24) 7.1, p =0.97	***r = 0.35; (n = 38) p = 0.04***
**Traditional Interview**	76.5% (n = 28) SD 10.4 95% CI 72.5-80.5%	Females (n = 23) 76.7%; Male (n = 5) 75.4%; p = 0.83	SEC 1& 2 (n = 23) 77.3%; SEC 3, 4, 5 (n = 5) 72.8%, p = 0.31	r = 0.13 (n = 28) p = 0.5
**GPA**	^^^Median out of max possible score of 4 was 3.9 (n = 45) min = 2.8, max = 4; interquartile range 0.3	Females (n = 36) 3.9, SD 0.3, Min 3, Max 4_ Males(n = 9) 3.9, SD 0.3, Min 2.8, Max 4_ Mann Whitney U p = 0.81	SEC 1&2 (n = 38) median GPA 3.9; SEC 3, 4, 5 (n = 7), median GPA 3.9, Mann Whitney U p =0.96.	Spearman’s rho −0.05, p = 0.73
**MMI**	67.1% (n = 109) SD 9.7, 95% CI 65.2-68.9	Females (n = 68) 66.9%; Males (n = 41) 67.3% p = 0.83	SEC 1&2 (n = 81) 67.1%; SEC 3, 4, 5 (n = 24) 66%, p = 0.66	r = 0.15 (n = 109) p = 0.12

*Significant results highlighted in bold italics.

### Outcome variables

Table 4 presents students’ scores on the overall First Medical Year Assessments and the OSCE. Cronbach Alpha of OSCE was 0.70. There was no significant difference between EU and Non-EU students in terms of mean First Med Overall Score however students who had English as a first language out performed those who did not (Difference in mean First Med Overall Score 3.4%; 95% CI 0.4- 6.3, p = 0.03). With respect to OSCE scores there was no significant difference in mean scores between EU and Non-EU students nor between those with English as a First Language or not.

**Table 4:** Outcome variables and relationship with gender, SEC, age, EU / Non-EU background and English as first language*

**Outcome variable**	**Mean Score, SD, 95% CI of mean**	**Gender**	**SEC**	**Age**	**EU versus Non-EU**	**English First Language Yes/No**
**First Med overall score**	65.5% (n = 109) SD 8.1, 95% CI 63.9-67	Female (n = 68) 65.46%; Male (n = 41)65.48%, p = 0.99	SEC 1&2 (n = 81) 65.4%; SEC 3, 4, 5 (n = 24) 65.8%, p = 0.82	r = −0.02; p = 0.84	EU students (n = 64) 66.3% SD 8.4; Non-EU students (n = 45) 64.2%, SD 7.5, p = 0.17	***First Language English (n = 70) 66.7% SD 8.5, First Language not English (n = 39) 63.3% SD 6.9***, ***p = 0.03***
**OSCE Chronbach alpha 0.70**	81.7% (n = 109) SD 5.1, 95% CI 80.7-82.6	***Females (n = 68) 82.8% SD 4.2; Males (n = 41) 79.9% SD 5.8 p = 0.007***	SEC 1&2 (n = 81) 81.9% SD 4.8; SEC 3, 4, 5 (n = 24) 81.3% SD 5.5, p = 0.64	r = −0.15; p = 0.03	EU students (n = 64) 81.5% SD 5.2; Non-EU student (n = 45) 82% SD 4.9, p = 0.61	First Language English (n = 70) 81.6% SD 5, First Language not English (n = 39) 81.8% SD 5.2, p = 0.83

*Significant results highlighted in bold italic.

### Correlations

Table 5 shows the correlations between selection data and the outcome variables for the whole sample. Significant positive correlations emerged between MMI and IELTS (large). When the sample were split according to origin (EU and Non-EU separately), a further significant correlation emerged between EU students’ MMI results and OSCE results (r = 0.27, n = 64, p = 0.03) (small).

**Table 5:** **Correlations between selection and outcome variables**

	**MMI**	**OSCE**	**First Med-Score**	**LCE**	**HPAT**	**IELTS**	**GPA**	**Trad-I**
**MMI**	_	.09	.11	-.07	.21	.50**	-.23 (rho)	.27
**OSCE**	.09	_	.33*	.19	-.25*	.02	-.10 (rho)	-.16
**First Med score**	.11	.33**	_	.52**	-.27* (rho -.36**)	.44**	.10(rho)	-.21
**LCE**	-.07	.19	.52**	_	-.28* (rho -.37**)	n/a^^^	n/a^^^	n/a^^^
**HPAT**	.21	-.25*	-.27* (rho -.36**)	-.28* (rho -.37**)	_	n/a^^^	n/a^^^	n/a^^^
**IELTS**	.50**	.02	.44**	n/a^^^	n/a^^^	_	.42*(rho)	.02
**GPA**	-.23 (rho)	-.13 (rho)	.10 (rho)	n/a^^^	n/a^^^	.42*(rho)	_	-.10 (rho)
**Trad-I**	.27	-.16	-.21	n/a^^^	n/a^^^	.02	-.10	_

Foot-notes:

All correlations calculated using both parametric and non-parametric tests. Pearson’s product moment correlation value (r) listed except where there were differences between the findings and in this case both Pearson’s r and Spearman’s rho reported. GPA was not normally distributed hence Spearman’s Rho is used throughout for this variable. Significant results highlighted in bold italics.

**Correlation is significant at the 0.01 level (2 tailed).

*Correlation is significant at the 0.05 level (2 tailed).

n/a ^^^Some correlations are not appropriate as students from the EU and Non-EU streams sat different selection tests-.

Please see List of Abbreviations.

### Regression analysis

A linear regression model was fitted separately for EU and Non EU students, initially with all predictors (full model) then used variable selection to identify potentially useful predictors. For EU students LCE was the only useful predictor (R^2^ 27%; p < 0.0005), while for Non-EU students GPA was the only significant predictor (R^2^ 53%; p < 0.0005).

### Qualitative results

Two overarching themes emerged. Authenticity describes participants’ views on the trustworthiness of MMI and factors that impacted on this in both a positive and negative way. Cultural Awareness captures participants’ understanding of how cultural values, beliefs and perceptions influenced both candidate and assessor performance at MMI. Quotes are identified as follows: Non-EU = Non-EU Student Focus Group; EU = EU Student Focus Group; MMI A1 = MMI Assessor Focus Group 1; MMI A2 = MMI Assessor Focus Group 2.

### Theme 1– Authenticity

#### Deeper understanding

Participants believed that selection “*is really high stakes. You either get into the career of your choice or you don’t*” (MMI A2). EU and Non-EU student reaction was overwhelmingly positive. They viewed MMI as a very authentic assessment of high value which was “*more thorough*” (Non-EUS) than alternative tools. MMI offered “*insight into how you cope and handle things that wouldn’t be apparent in a regular interview*” (Non-EUS) and provided “*more of a chance to show who you were*” (EUS). As one candidate stated “*I kind of felt that I was really being forced to think on my feet*” (EUS). There was broad consensus amongst students that MMI was a welcome addition to selection.

Assessors viewed the main benefit of MMI was the opportunity to “*get a feel for the person themselves and how that could translate into the [medical] course”* (MMI A1). It provided “*a deeper understanding”* of candidates (MMI A1). One assessor viewed the purpose of MMI to “*..make sure people who are totally not suitable for medicine don’t go into it rather than you know ranking people who are good*” (MMI A2). In comparison with other selection tools, assessors felt that MMI was “*better than the HPAT-[Ireland]*” (MMI A2) and “*certainly better than just academic performance*” (MMI A2).

#### Relevance to clinical practice

A big advantage was that MMI station content was seen as “ very *relevant*” (EU) to “*the skills that we need to have when we are doctors*” (Non-EU) and in this way students felt it offered authentic insight into “*what you’re going to be doing ten years down the line*” (EU).

An unexpected advantage of this was that students felt primed towards the skills that were important for them to develop in their undergraduate career: “ *I feel that MMI actually help[ed] us in developing those skills*” (Non-EU). A small number of assessors also viewed this “*formative”* (MMI A2) role important especially with respect to students’ “*communication skills*” (MMI A2) and “*professionalism*” (MMI A1).

Assessors felt that preparation for the MMI stations ensured that students “ ..*really had a better feel for what …. the day to day working as a medical doctor is. It actually forces them to put themselves in those situations*” (MMI A2).

#### Reservations and recommendations

Assessors expressed more reservations than students. A number of assessors (approximately 3) were concerned that MMI felt “*like a bit of a performance*” (MMI A1). They worried that students “*were trying to work out what*” was expected from them and “*give you exactly that”* (MMI A1). Coaching was seen as a threat that could “*undermine the whole process*” (MMI A2) and reduce its ability to be “*discriminatory “* (MMI A1). Commercial coaching could have a negative effect, because of the associated cost: “*it’s just you end up with a lot of people from the highest socio-economic classes*” (MMI A2). Students suggested that live MMI stations be set up on medical school “*open days”*(EU) where applicants could practice and that sample MMI stations be available “*online so that people could look them up if they couldn’t afford to go to a [coaching institution*]” (EU).

Some assessors admitted “*responding more to the candidates who were confident humorous and warm”* (MMI A1). This led to a concern that “*students who are more nervous…. a little bit shyer*” (MMI A1) or “*less empathic*” (MMI A2) may be “*negatively discriminated against*” (MMI A1) by MMI. Some students also voiced this concern and the possible impact on selection

“*if you have very good communication skills you can do very well on the MMI.. So it’s almost singling out that group of people”* (EU).

It was suggested to “*broaden the range of skills or attributes*” (MMI A1) assessed to counter the perception that MMI over emphasised “*communication skills and empathy*” (MMI A1). For example by including stations that tested “*efficiency and problem solving*” (MMI A1), that could identify applicants who were “*dexterous, skilled and imaginative*” (MMI A2) and that did not penalise applicants who were “*a bit brisker in their communication styles*” (MMI A1).

Other reservations included recognition that MMI was “*time consuming … labour intensive*” (MMI A1) and “*very costly*” (MMI A2) to run and concern about using MMI for entry from second level education

“*These are very young students. We are bringing them into college to teach them some of these skills….. Is this the right time to be assessing it? I’m not sure*” (MMI A2).

### Theme 2– cultural awareness

#### Culture, attitudes and station content

Assessors and students observed that “*the cultural context was an issue through a number of the stations*” (MMI A1). Culture and attitudes were particularly relevant in stations where societal matters which were “*subtle and culture specific*” (MMI A1) such as when a student with an alcohol problem or organ donation were discussed. (See Additional file 1: Appendix 1).

“*I know the, Islam[ic] students in our class in particular would have been very taken aback by the alcohol station …*” (EU).

International students clearly recognised the challenge culture posed to their communication skills:

“*I couldn’t put myself in her shoes …..it is so difficult because our cultures are different so I can’t put empathy there*” (Non-EU- reflecting on the alcohol station).

Conversely one international student saw merit in challenging applicants’ cultural views:

“*drinking is not in our culture, so I think that situation is a must in selection tools because we can see, …..how they cope with the issue that is outside of their own life*” (Non-EU).

One solution was to use stations based on “*behavioural things that cut across cultures*” (MMI A2) such as “*cheating… for breaches of professionalism*” (MMI A2) which may be more accessible to all applicants. An alternative suggestion was “*… to bring in some diverse cultural issues into it, like witchcraft… things that are not the typical things you’d meet in Ireland*” (MMI A2). These stations would then prove equally challenging to host country and international applicants.

Another common suggestion for improvement was to increase the standard and scope of the information that was made available to applicants in advance of the MMI. Knowledge “*of the kinds of station [to expect]”* (Non-EU) would allow students to “*prepare beforehand*” (Non-EU).

#### Culture and assessor subjectivity

Assessors were acutely conscious of the link between culture and assessor subjectivity:

*“I’m assessing from my viewpoint, cultural viewpoint which can be very, very different to others and I would think that I’m probably not a very* reliable [assessor]” (MMI A2).

Culture impacted significantly on how assessors viewed candidates’ interpersonal communication. This was evident both in the stations with a role player:

“*Different cultures comfort people in different ways and so my perspective of someone comforting a friend, it’s completely different to some international students who sat beside them and had their arm around them, which is culturally acceptable in their cultures..*” (MMI A2).

And the one to one interview type stations:

“[in the ] *one to one interviews…..some people discuss things with humour but humour doesn’t necessarily translate…. so then it just came off as weird… you could lose all of your marks.*” (MMI A2).

Detailed assessor training on cultural awareness was seen as essential;

“…*if I’m not informed of a different person, different culture, cultural practice, how can I assess them reliably?”* (MMIA2).

So too was recruiting *“multiple examiners from different cultures”* (MMIA2). In addition assessors strongly recommended that MMI *“pilots”* (MMI A2) should be part of normal advance MMI preparations and should include students from a variety of cultural backgrounds to ensure *“a representative core…of the core sitting the exam*” (MMI A2).

#### English language proficiency

International students recognised the important influence English language proficiency had on performance at MMI “*if you didn’t understand the questions how are you going to perform very well?*”(Non-EU). They also observed that “*not having it[English] as your first language …could make you more nervous”* (Non-EU) which again could impact negatively on performance.

Assessors too considered English language proficiency “*a very big barrier*” (MMI A1). However some EU students felt that the MMI was “*a useful screening tool for the English language*” (EU) which was important because

“….*if they can’t understand the MMI, it’s going to be very difficult for them to understand lectures and therefore they’re not going to do well in Ireland*” (EU).

A contributing factor was that “*the time limitation is really short*” (Non-EU) and students recommended lengthening the allotted time.

## Discussion

The reputation and use of MMI as a selection tool for medicine is growing [11]. At the same time migration patterns of medical students internationally continue to rise. This study aimed to establish the fairness, predictive validity and stakeholder acceptability of MMI in an internationally diverse student population.

In this sample MMI performed as well, or better than, the other selection tools in terms of fairness with respect to age, gender and socioeconomic class (see Table 2). We found no evidence that performance on any of the selection tools was influenced by the SEC of the candidates although this has been demonstrated elsewhere [12,42]. It is possible this may be a type 2 error due to our small sample size.

Widening diversity to medicine has become an important consideration in the choice and on-going use of selection tools [6,43]. *Equal opportunity* is an important aspect of distributive justice. As MMI becomes more widely used it is likely that commercial coaching will become more prevalent, which may negatively impact lower socioeconomic applicants; a concern raised by our assessors. As suggested in the focus groups medical schools could mitigate this by providing open-source online access to preparatory materials, including mock stations and marking grids, links to resources outlining the relevance of MMI stations to clinical practice and guidelines for professional behaviours and standards in medicine. Ensuring that essential cultural and linguistic information was included in this resource would help extend equal opportunity to international applicants. These and other recommendations from the study have been summarised in Table 6.

**Table 6:** **Best practice recommendations**

ᅟ	ᅟ
1.	Consider what domains to test and blueprint these against the relevant medical school and nationally agreed learning outcomes, regulatory standards and on the job requirements
2.	Ensure adequate diversity of domains tested to avoid over reliance on any one skill set
3.	Consider cultural issues in the design and development of stations. Opt for culturally neutral material or adequately diverse cultural issues to avoid giving an advantage to any one group of candidates.
4.	Pilot stations with candidates and assessors from a range of cultures, where possible mapped to the cultural backgrounds of the relevant applicant pool
5.	Provide adequate cultural awareness training for assessors and recruit assessors from a range of cultural backgrounds
6.	Use clear unambiguous language, avoiding colloquialisms, for candidate and assessor instructions and role players’ script
7.	Ensure station duration provides adequate time for candidates who are being assessed in a language other than their first language
8.	Provide free preparatory information to applicants in advance via a variety of media – e.g. Medical School Open days, online and printed materials. Material should include sample stations, a description of the MMI process, justification for the range of domains tested in terms of job relatedness, links to professional standards and medical school learning outcomes.
9.	Regularly audit applicant and successful candidates for demographics including age, gender, socioeconomic group and cultural backgrounds to monitor for fairness.
10.	Monitor for evidence of predictive validity on an ongoing basis.
11.	Provide adequate supports and formative feedback to international students throughout their training
12.	Draft globally agreed minimum standards for selection processes

The predictive validity of MMI with respect to First Med assessments was weaker in this study than reported predictions of medical course assessments elsewhere but somewhat better in terms of prediction of OSCE scores of EU students [44]. Why MMI was more predictive of EU rather than Non-EU students’ OSCEs is unclear. The First Medical Year in NUI Galway is largely devoted to the pre-clinical sciences and knowledge based assessments; these observed predictive validity patterns may change as students progress through the course and spend more time in the clinical domain.

Recently there has been significant controversy over evidence that IMGs perform less well than home graduates in several higher professional training examinations in the UK, USA and Canada [12-14,45,46]. In light of this controversy a challenging finding from this study was that Non-EU students’ mean MMI score was significantly below that of EU students. The same pattern emerged for students without English as a first language. In terms of *equity of outcome* these data require detailed consideration. Arguably selection is the most significant exam in one’s medical career hence understanding the mediating and moderating influences of these finding is important. It assumes even greater importance should both home and international applicants be competing for the same places.

Similar proportions of EU and Non-EU students volunteered to participate in this study and there is no evidence to suggest that levels of preparation or motivation were lower amongst international students. The fact that EU and Non-EU students did not differ in First Med Overall Score, or OSCE score implies that levels of ability in both groups was similar, at least by the end of Year 1 and rules out differences in cognitive ability as a likely explanation. The quantitative and qualitative data however support the centrality of culture and language to MMI performance. Comparing MMI with currently used selection tools highlighted that the strongest association, a significant large strength correlation, lay between MMI and English language ability, with higher IELTS scores associated with enhanced MMI scores. Ireland uses a minimum IELTS subsection and overall score of 6.5 for entry which is a point lower than many UK schools and may go some way to explain the discrepancy in MMI scores [14]. Tiffin et al. have established that IELTS scores significantly predict IMGs’ clinical competence as measured by performance on the Annual Review of Competence Progression: each IELTS point scored above 7 increased the odds of achieving a more satisfactory appraisal by 69% [13].

English language proficiency was seen as a very significant barrier by both assessors and students. Apart from simply raising the eligible IELTS entry score, which may have the undesirable effect of disqualifying some potentially good applicants, a number of measures could be taken to improve the characteristics of the test (see Table 6). Further work is required to establish if these measures would have a beneficial effect in reducing the language hurdle. If shown to be helpful then these measures may enhance the procedural and ultimately distributive justice of the MMI. The EU student focus group recognised that English language proficiency is also associated with ability to understand lectures and perform as a medical student. This was supported by quantitative data showing that First Med Overall mean score for students with English as a first language was over 3% higher than those without. This highlights the duty of responsibility medical schools have to provide adequate monitoring, feedback, support and access to additional training in conversational and medical English for non-native speakers.

Issues of language were closely aligned with culture and assessor subjectivity. Assessors were particularly concerned that the cultural context of some of the MMI stations, or their marking of them, may be unfair and potentially diminish *equity of outcome* for international students. They suggested additional training in cultural sensitivity. Cultural sensitivity means being aware that cultural differences and similarities exist and have an effect on values, learning and behaviour [47]. Cultural training is the norm for examiners on many postgraduate training programmes [48]. In addition to training in the design of culturally sensitive stations assessors recommended purposefully recruiting assessors from different backgrounds and involving international students in the piloting of MMI stations. All of these measures increase the resources and cost of MMI but in terms of best practice guidelines for MMI developers they are worthy of due consideration.

This study focused on the selection of students from an international background. However the issues raised with respect to culture are broadly pertinent to selection as a whole. Approximately 30% of “home” UK medical students (those with UK nationality) are from ethnic minorities and applicants from these backgrounds may be equally as challenged by some of the culturally specific stations [49]. As the use of MMI becomes main stream it is important to ensure that ongoing audit and evaluation of the performance of both international applicants and those from different cultural and ethnic minority backgrounds is undertaken and made publically available.

In our study stakeholders’ perceptions of the procedural justice of MMI were enhanced by its relevance to future clinical studies and the adequate opportunity it provided to candidates to demonstrate their ability. Non-EU students highlighted that the MMI experience primed them for the skills they would need to acquire in their undergraduate education and likewise assessors saw an important role for MMI in giving formative feedback. It could be argued that there is a responsibility on medical schools to ensure that information outlining successful candidates’ strengths and weaknesses is fed back to them to guide their learning. This would be particularly relevant for issues of professionalism, culture, attitude and language. Says et al. have piloted the use of MMI in Saudi Arabia and, although MMI is not yet a formal part of the selection process, they use it to identify both outstanding and below average performing students targeting the latter for additional supportive workshops [50]. This is a model that could be applied elsewhere and likely to be welcomed by students.

Study limitations include that this is a single study, situated in one medical school with a small sample and one year of follow-up. It is possible that our findings were influenced by other confounding variables within the MMI, or systematic examiner bias. Students were already selected to medicine and along with assessors they volunteered to take part. This self-selected group may differ in meaningful and underdetermined ways to their counterparts. A further limitation is that this MMI was experimental hence the usual pressures, motivations and preparation undertaken by candidates in advance may not have applied in this circumstance. By contrast the main strength of this study is that the methodology provided for a rich and deep understanding of both the numerical data and facts relating to MMI as well as the meaning it had for both assessors and students.

Further considerations - Recognising the growing patterns of migration in medical school applicants and the heterogeneity of selection practices internationally perhaps the time has come to draft globally agreed minimum standards of selection practice similar to those for medical education programmes [51]. International medical students are an important source of revenue for Medical Schools. They are however not a homogenous group. This study highlights the duty of Medical Schools in terms of social responsibility, to ensure that international students are provided with adequate supports, particularly with respect to culture and language, tailored to their individual needs. In these circumstances MMI could provide helpful formative feedback.

## Conclusions

In conclusion MMI has proved a welcome addition to assessment armamentarium. This study found that MMI demonstrated good job relatedness and acceptability, particularly amongst candidates. Understanding the mediating and moderating influences of differences in performance of international candidates is essential to ensure that this selection tool complies with the metrics of good assessment practice and principles of both distributive and procedural justice for all applicants, irrespective of nationality and cultural background.

## Additional file

Additional file 1:
**Appendix.**

## Abbreviations

CAO: Central Applications Office
GPA: Grade Point Average
HPAT-Ireland: Health Professions Admission Test Ireland
IELTS: International English Testing System
IMGs: International medical graduates
LCE: Leaving Certificate Examination
MMI: Multiple Mini Interview
OSCE: Objective Structured Clinical Examination
Trad I: Traditional Interview
