# Supplementary material for: The fairness, predictive validity and acceptability of multiple mini interview in an internationally diverse student population- a mixed methods study
Source: BMC Med Educ. 2014 Dec 21;14:267. doi: 10.1186/s12909-014-0267-0 (PMC4302428; doi:10.1186/s12909-014-0267-0)
Supplement: Additional file 1: — Appendix. [file 12909_2014_267_MOESM1_ESM.docx]

**Additional File 1**

**Appendix 1 MMI Description**

There were ten MMI stations in total each lasting seven minutes, with one examiner per station. Station content was provided by Dundee Medical School. Modifications to station content were made where necessary to ensure authenticity in an Irish setting. Five stations involved an interviewer, a role-player and the candidate. The other five stations were interview based (one interviewer: one candidate).

Each station was scored across three domains and one global rating scale. Domain scores ranged from 0-5 (0= poor; 5 = excellent) with detailed written descriptors of excellent and poor performances. Global score were on a five point scale ranging from unacceptable to excellent performance.

|  | **Station Content** | **Mapped to Irish Medical Council Eight Domains of Professional Practice** |
| --- | --- | --- |
| 1 | Counselling conversation with a distressed medical student | **Relating to patients**  Management |
| 2 | Discussion of possible breaches of professionalism by a medical student | **Professionalism**  Communication and Interpersonal skills |
| 3 | Conversation with a medical student who was drinking alcohol to excess | **Relating to patients**  Professionalism |
| 4 | Interview about motivation and preparation to study medicine | **Scholarship**  Communication and Interpersonal skills |
| 5 | Candidate and helper complete a puzzle station | **Collaboration and Teamwork**  Relating to patients |
| 6 | Interview about the experience of team work, achievements and social responsibility | **Scholarship**  Communication and Interpersonal skills |
| 7 | Discussion about what makes a fair health care system | **Patient safety and quality of patient care**  Professionalism |
| 8 | Giving advice to a non-English speaking patient | **Communication and Interpersonal skills**  Relating to patients |
| 9 | Discussion about the issues relating to organ donation | **Professionalism**  Communication and Interpersonal skills |
| 10 | Completing a complex card sort with a helper | **Management**  Collaboration and teamwork |

**Appendix 2**

**Description of First Year Medical Course/ Outcome Variables**

**First Year Modules and Assessments**

The medical undergraduate degree programme is an integrated modular, systems based curriculum. The First Year Medical Course comprises 10 modules which together equal 60 ECTS. The modules and ECTS credit weighting are listed below. In general knowledge based assessments (eg MCQs/ written papers) account for 70% of the mark per module, while practical / skills based assessments (eg laboratory exams/OSCEs) account for 30% of the mark.

**Semester 1**

Musculoskeletal System and Peripheral Nerves 10 ECTS

General Principles of Human Body Structure 5 ECTS

Principles of Physiology 5 ECTS

Biomolecules, Metabolism and Energy 5 ECTS

Medical Professionalism (1) 10 ECTS (This module includes ethics, law, health promotion, health informatics, communication and clinical examination skills and a special study module).

**Semester 2**

Cardiovascular System 5 ECTS

Respiratory System 5 ECTS

Renal System 5 ECTS

Gastrointestinal System 5 ECTS

Metabolism, Nutrition and Health 5 ECTS.

**Details of the outcome variables:**

**First Med Score** was a continuous variable representing each student’s percentage score overall across all first year modules. It is calculated based on their percentage mark out of 100 for each of ten modules and is adjusted to reflect the relative weightings of each of the module in terms of its allocated credits according to the formula: Sum of (Percentage mark* credit weighting of module) / total number of credits).

**First Med OSCE** score, a continuous variable representing performance on a five station OSCE that contributes (one third) to the professionalism score. The OSCE stations examine communication and clinical skills and are of 5 minute duration. The OSCE is conducted in two parallel cycles, with one examiner and one simulated patient per station, per cycle. OMIS software^1^ is used to mark the OSCE stations online. OSCE marking is based on a combination of a checklist and clinical / communication descriptor bands. The Calgary Cambridge Model^2^ forms the basis of the communication skills checklist.

Example OSCE stations are

1. Asking a patient for consent to measure vital signs, measuring them and explaining the findings to the patient.
2. Performing urinalysis on a sample of urine and explaining the findings to the patient.
3. Measuring a patient’s blood pressure and discussing the results with the patient.
4. Calculating a patient’s BMI and discussing results.
5. Performing Basic Life Support and using an automated external defibrillator on a mannequin

1 Kropmans TJB, O’Donovan BGG, Cunningham D, Murphy AW, Flaherty G, Nestel D, Dunne FP “An Online Management Information System for Objective Structured Clinical Examinations”. Computer and Information Science 2012; 5; 1

2. Silverman, Jonathan, Suzanne M. Kurtz, Juliet Draper, Jan van Dalen, and Frederic W. Platt.*Skills for communicating with patients*. Oxford, UK: Radcliffe Pub., 2005.

**Appendix 3 FOCUS GROUP TOPIC GUIDE**

**Topic 1: Exploration of General Thoughts**

Key questions: What are your thoughts having examined at / sat the MMI?

Specific probes: From your experience how do you think MMI compares to other tools used for the selection of medical students?

**Topic 2: Potential role for MMI in the selection of medical students in Ireland**

Key questions: Do you think the MMI has a potential role in the selection of medical students in Ireland?

Specific probes: What do think the barriers to its use might be? What advantages might it offer over current selection tools? Do you have any suggestions that might make MMI a viable option for selection in Ireland?

**Topic 3: Establishment of general views on the use of MMI for selecting students from international backgrounds**

Key Questions: What are your views on the utility / appropriateness of MMI as a selection tool for medical students from international backgrounds?

Can you expand on this? / Do you see any advantages to using MMI in this context? / Do you see any barriers to using MMI in this context?

How do you think MMI would compare to traditional interview in the selection of international students?

**Topic 4: Impact of English language proficiency on MMI performance (If not already explored in Topic 2)**

Key Questions: To what extent do you think English language proficiency can impact on a candidates’ performance in the MMI? Can you expand on this?

**Topic 5: Impact of cultural issues on MMI performance (If not already explored in Topic 2)**

Key Questions: To what extent do you think cultural issues could impact on a candidates’ performance in the MMI? Can you expand on this?

(If the interviewee is unsure of what is meant by this then prompt with- for instance one of the MMI stations referred to a medical student drinking alcohol to excess- this may be a particular issue for Irish medical students and as such is quite culturally specific).

**Topic 6: Recommendations for best practice**

Key Questions: Can you suggest any recommendations for designing and running MMIs that would be appropriate to use in the selection of medical students from international background?

Specific Probes:

In the development of station content? / In the running of the MMI? /

**Final Questions:** Is there anything else you would like to say about the MMI?

Is there anything I haven’t asked you that you feel I should have?
